# Clinical comparison of laparoscopic and open surgical approaches for uterus-preserving myomectomy: a retrospective analysis on patient-reported outcome, postoperative morbidity and pregnancy outcomes

**DOI:** 10.1007/s00404-024-07818-2

**Published:** 2024-11-27

**Authors:** Lucia Anna Otten, Subhiyeh Lama, Jakob Wilhelm Otten, Kira Winkler, Damian Johannes Ralser, Eva Katharina Egger, Mustea Alexander

**Affiliations:** 1 Clinic for Gynaecology and Gynaecological Oncology, Uniklinikum Bonn, Venusberg-Campus 1, 53127 Bonn, Germany; 2 Gastroenterology, Hepatology, General Internal Medicine, Helios Klinikum Siegburg, Ringstraße 49, 53721 Siegburg, Germany; 3 Clinic for Oral, Maxillofacial and Plastic Facial Surgery, Uniklinikum Bonn, Venusberg-Campus 1, 53127 Bonn, Germany; 4 Department of Senology, Uniklinikum Bonn, Venusberg-Campus 1, 53127 Bonn, Germany

**Keywords:** Myoma, Uterine fibroids, Minimal invasive surgery, Gynecology

## Abstract

**Purpose:**

Uterine fibroids pose clinical challenges due to varied symptoms and impact on fertility. Aim of this research is to compare open and laparoscopic myomectomy, with focus on evaluating their effects on patients' quality of life and analyzing their implications for pregnancy outcomes.

**Methods:**

This retrospective study compares open and laparoscopic myomectomy outcomes in 168 patients treated October 2017 and July 2023. Preoperative characteristics and postoperative outcomes in terms of symptoms and pregnancy outcomes were examined.

**Results:**

The patient cohort comprised patients with a high symptom burden. Only 51.2% expressing a desire for future pregnancies, highlighting diverse motivations for uterus-preservation. No significant differences were observed in preoperative symptoms. Larger and multiple myomas were associated with a higher likelihood of laparotomy. Recurrence rates were lower after laparoscopy (10.2% vs. 23.8%, *p* = 0.02). Cesarean section recommendations were more frequent post-laparotomy group (36.6% vs. 86.6%, *p* = 0.000). Morbidities and satisfaction showed no significant differences between approaches, with slightly better bleeding improvement after laparotomy. Despite similar pregnancy outcomes, a high proportion of patients did not conceive postoperatively (75.4%). Among patients who became pregnant postoperatively (*n* = 31), most patients conceived after one year or more, with no dependence on the surgical approach (*p* = 0.227).

**Conclusion:**

Both open and laparoscopic myomectomy surgeries showed high patient satisfaction, symptom alleviation, and comparable pregnancy results. A preference emerged for laparoscopy in terms of cesarean section recommendations and recurrence risk. Laparoscopic procedures tended to offer higher operative satisfaction and fewer complications. The study emphasized the complexity of therapeutic decision-making.

## What does this study add to the clinical work

**Table:** 

Both open and laparoscopic myomectomy surgeries showed high patient satisfaction, symptom alleviation, and comparable pregnancy results. A preference emerged for laparoscopy in terms of cesarean section recommendations and recurrence risk.	

## Introduction

Uterine fibroids, or myomas, represent the most prevalent benign tumors affecting women. They occur in up to 80% of women before the age of 50 [1]. With advancing age, there is often an increase in size until menopause, followed by a subsequent reduction in size, as their growth is hormonally dependent. Myomas originate monoclonally from a single cell within the muscular layer of the uterus. Risk factors include African descent, early menarche, nulliparity, obesity, positive family history of myomas, hypertension, alcohol consumption, and high intake of soybeans [1–3]. Protective factors include late menarche, high parity, and smoking [4].

Depending on the location and number of myomas, symptoms range from asymptomatic to severe complaints such as pain, menstrual irregularities, pressure on the bladder, bowel, and ureters, as well as dyspareunia. However, only 30% of women with myomas experience symptoms [1, 2]. The classification of their location is based on their position (subserosal, submucosal, or intramural/Fédération Internationale de Gynécologie et d'Obstétrique (FIGO) classification) [5].

Primary diagnostic evaluation is carried out through ultrasound, often it is a diagnosis per incidence. In cases of large or multiple myomas, MRI may be useful for surgical planning in specific instances. Besides symptoms, myomas can impair conception and pregnancy, leading to preterm birth, increased cesarean section risk, postpartum bleeding, fetal malposition, intrauterine growth restriction (IUGR), and pain [4, 6–8, 17, 18].

For those with completed family planning, various conservative approaches (Reyqo (Relugolix + Estradiol + Norethisteronacetat), Hormonal IUD, embolization, HIFU, thermoablation (Sonata) [1, 2, 7–9, 12–16] can be considered, alongside the option of hysterectomy. However, in cases of desire for future fertility, uterine preservation is pursued. Procedures like embolization, thermoablation, and HIFU can be performed, though safety data during pregnancy are limited [9–13]. Myomectomy, through laparotomy, laparoscopy, or hysteroscopy, depending on location and size, is the standard procedure for those desiring fertility. While laparoscopic and hysteroscopic approaches exhibit lower morbidity, operational challenges may arise with larger lesions. Additionally, concerns regarding potential impaired wound healing of uterine incisions in laparoscopy need consideration.

Evidence suggests improved pregnancy rates post-myomectomy and reduced pregnancy complications [4, 20, 21], although surgery-related complications, such as uterine rupture, remain a concern [14].

The objective of this study is to compare open and laparoscopic myomectomy, specifically examining their impact on quality of life through patient-reported outcomes and assessing their influence on pregnancy. This includes considerations for the choice of delivery mode, protective factors, and risks influencing the therapeutic course.

## Methods

This study analyzes retrospective data extracted from patient records, as well as follow-up information obtained through standardized questionnaires sent to the patients. The course of the disease was anonymously collected from the digital records of the patients. Cases were identified from the hospital documentation system of the Department of Gynecology and Gynecologic Oncology at the University Hospital in Bonn using OPS codes (5–681.8 and 5–681.9) between October 2017 and July 2023. A total of 388 records were initially identified, and after removing duplicate patients and those not meeting the inclusion criteria or not reachable for follow-up, 168 patients were included.

Patients who underwent a different interventional myomectomy, hysteroscopic myomectomy, or HIFU, as well as those in whom adenomyosis or sarcoma was identified instead of a myoma during the course, were excluded. Each patient signed a preoperative consent and data protection declaration for participation in the study.

Two groups were compared: laparoscopically operated patients and those operated on openly. Preoperative characteristics and risk factors (age, number and location of myomas, size of the largest myoma, Fédération Internationale de Gynécologie et d'Obstétrique (FIGO) classification, bleeding disorders, pain, dyspareunia, constipation, pregnancies before surgery) and perioperative findings (hospital stay, scar size, complications, recommendation for cesarean section due to the surgery) were collected. Parameters were gathered related to subsequent pregnancies (number, time to conception) and the quality of life (postoperative restrictions, wound healing disorders, symptom improvement, cosmetic satisfaction, recurrences) of the patients, as well as overall satisfaction with the intervention. In addition to data from the patient records, information was obtained based on a questionnaire sent to the patients or collected during a telephone interview (see Appendix).

All questionnaires were be consolidated into a dataset in SPSS and statistically analyzed. The statistical analysis of patient data was conducted using Microsoft Excel and IBM SPSS Statistics (Version 29.0.1.0). Descriptive statistics included frequency, percentiles, and mean values. The statistical significance level was set at *p* < 0.05. Results were graphically presented in the form of box plots.

To analyze relationships between categorical variables, contingency tables were created using the Pearson Chi-Square test. Subsequently, the Linear-by-Linear test was employed for the further analysis of continuous variables, and the Likelihood Ratio test was used for non-continuous variables. The non-parametric Kruskal–Wallis test was used to examine differences and relationships in independent groups for ordinal and metric data.

The study proposal was reviewed and approved by the Ethics Committee of the University Hospital Bonn (reference number: 2024-40-BO).

## Results

### Preoperative patient characteristics

**Fig. 1:**
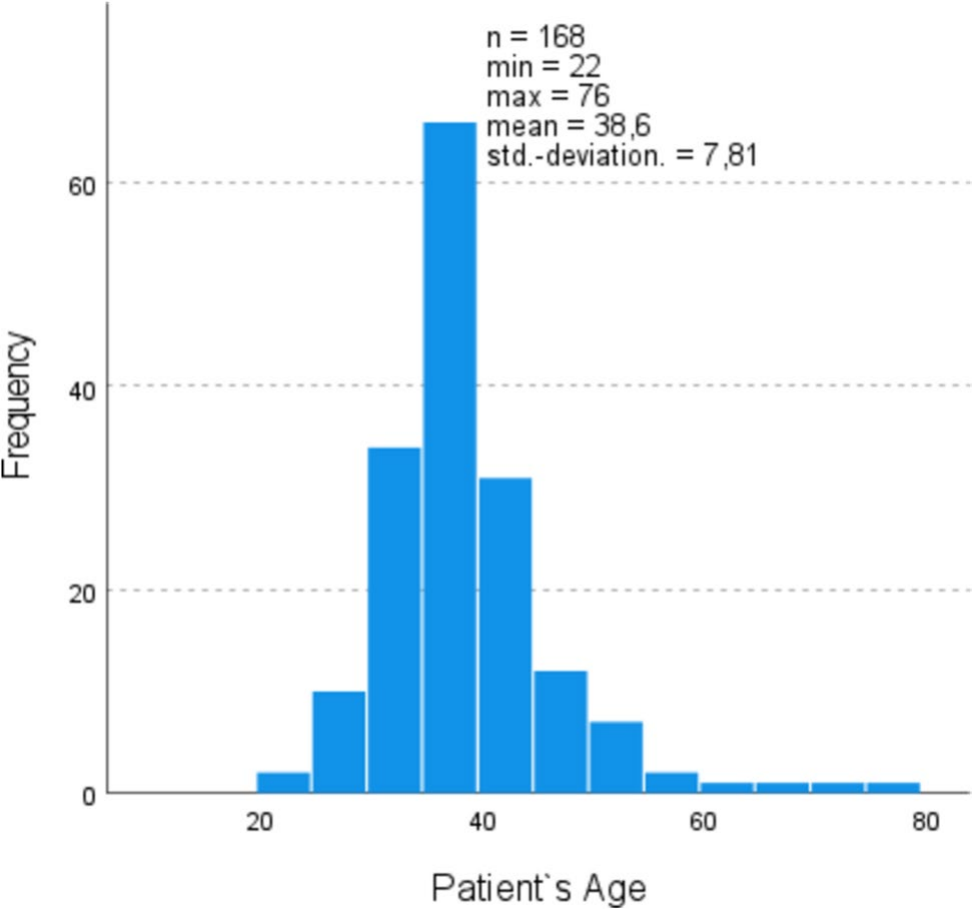
Age distribution of myoma

The patients ranged in age from 22 to 76 years, with an average age of 38 years. Only a small proportion were over 50 (7.8%). Regarding age, there was no significant difference between the two groups (*p* = 0.059) (Fig. 1).

In terms of preoperative symptoms, 42.9% reported normal to moderate bleeding, 24.4% reported heavy bleeding, and 28.0% reported very heavy bleeding. There was no significant difference between the two groups in bleeding severity (*p* = 0.225).

For preoperative pelvic pain, 25.0% reported no pain, 2.4% very mild pain, 14.9% mild pain, 13.1% moderate pain, 14.3% severe pain, and 24.4% very severe pain. No significant differences were observed between the groups (*p* = 0.9).

Regarding pain during intercourse, 73.8% reported none to mild pain, 13.1% moderate pain, 7.1% severe pain, and 3.0% very severe pain. There was no significant difference between the groups (*p* = 0.216).

For dysmenorrhea, 50.0% of patients reported no or very mild pain, 21.0% reported mild pain, 17.9% reported moderate pain, and 6.5% and 2.4% reported severe or very severe pain, respectively. No differences were observed between the groups (*p* = 0.766).

Regarding bladder pressure, 68.5% reported no to mild pressure, 19.6% reported moderate pressure, 1.8% reported very strong pressure, 7.1% reported strong pressure, and 3.0% reported very strong pressure. There was no significant difference between the groups (*p* = 0.139).

Digestive problems were reported by 64.9% of patients, with 14.9% experiencing mild discomfort, 13.7% experiencing moderate discomfort, 1.8% experiencing rather severe discomfort, and 4.2% and 0.6% experiencing severe or very severe discomfort, respectively. No differences were observed between the groups (*p* = 0.848).

Regarding preoperative pregnancies, 78% of patients had no children, 13.1% had one child, 6.5% had two children, and 1.8% had three children; one patient had four children. There was a significant difference between the groups (*p* = 0.005). Patients in the laparotomy group more often had no prior pregnancies (89.6% in the laparotomy group vs. 70.3% in the laparoscopy group). Among the patients, 51.2% expressed a desire for children preoperatively, with no difference between the groups (*p* = 0.174).

### Myoma characteristics

**Fig. 2:**
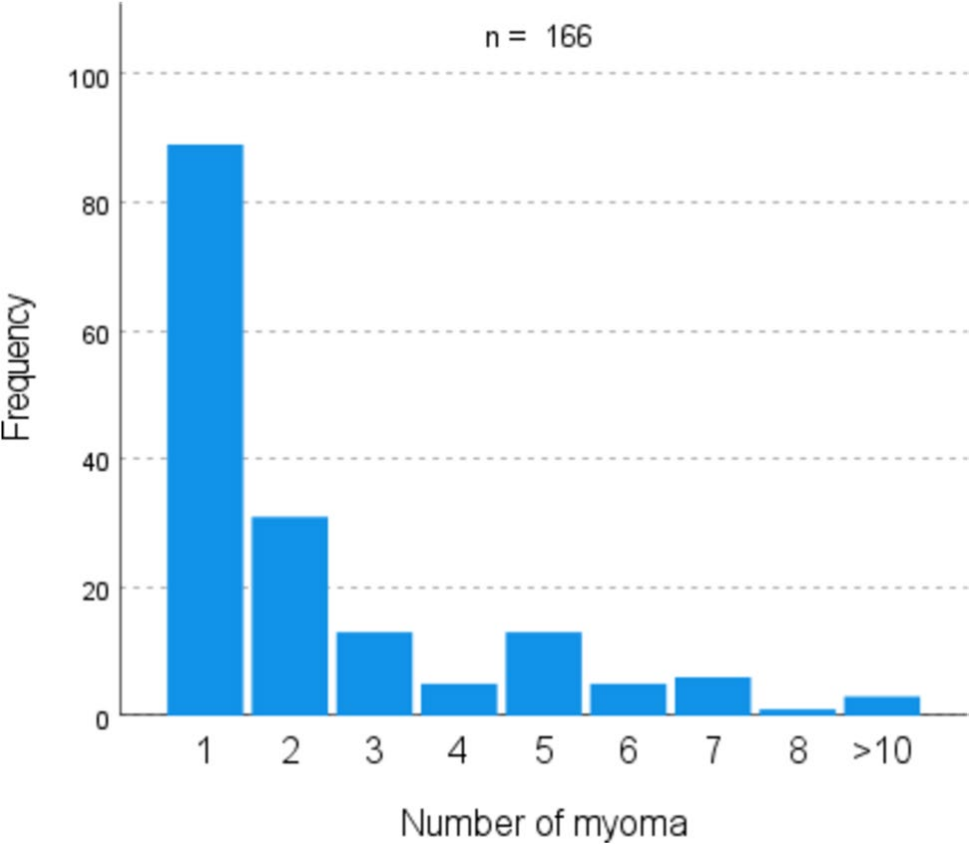
Number of myomas

**Fig. 3:**
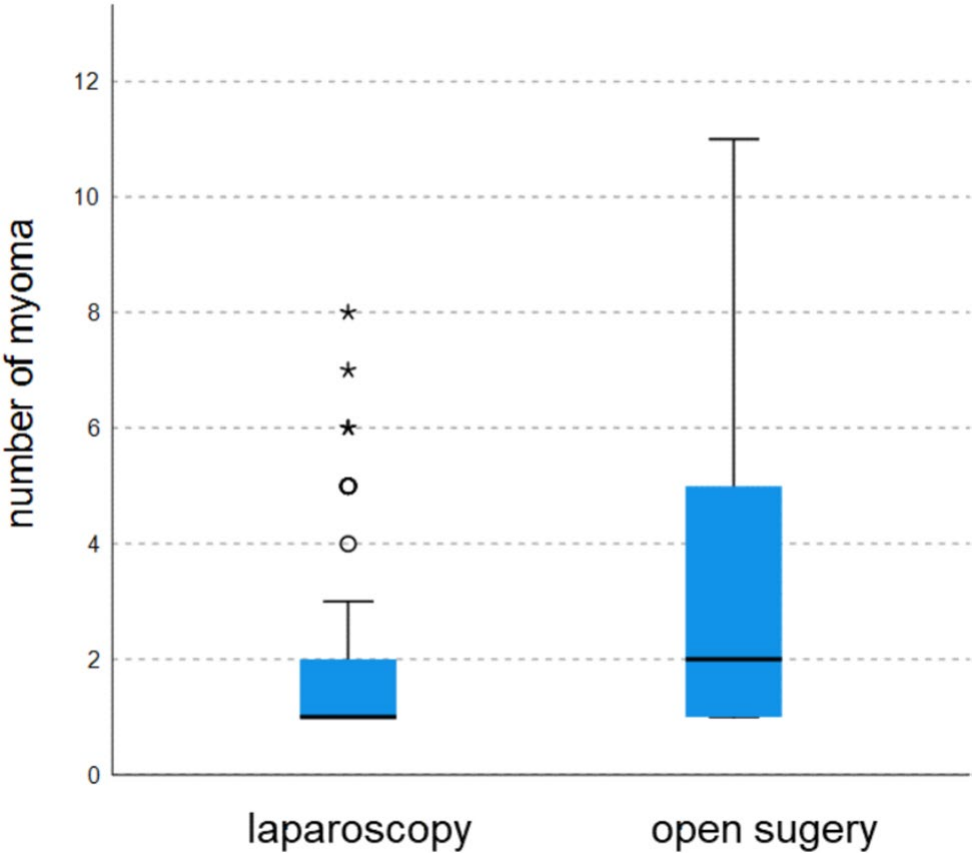
Distribution of myoma count laparoscopic versus open surgery

**Fig. 4:**
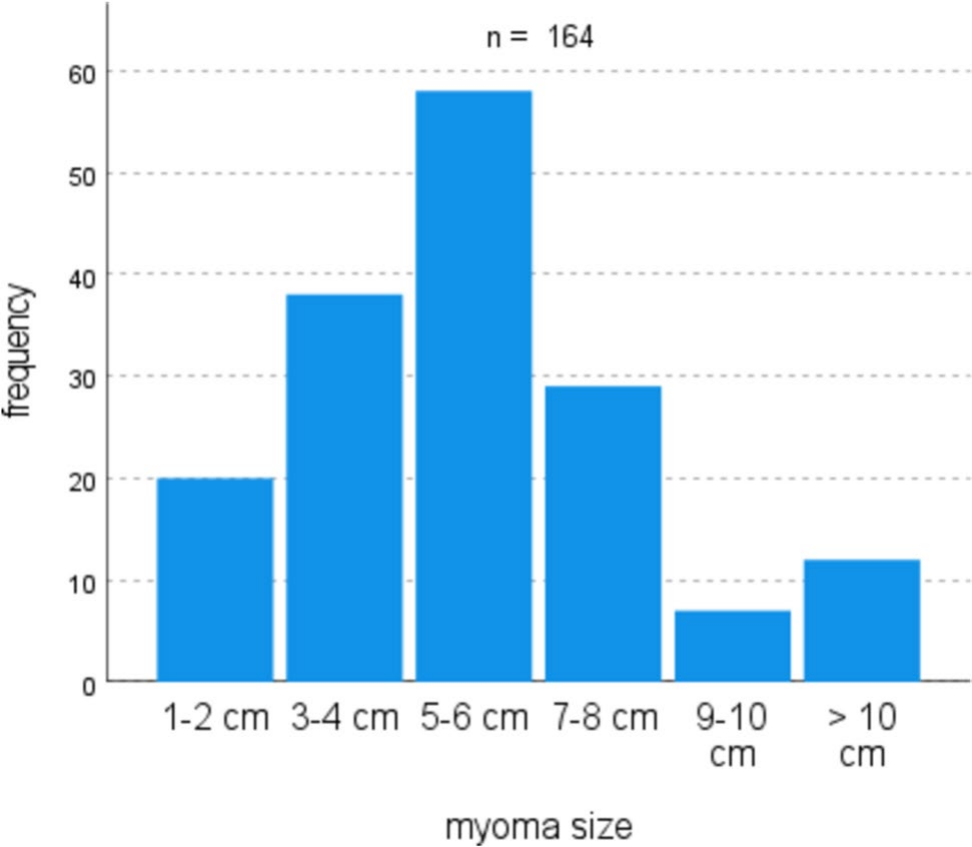
Distribution of myoma size

**Fig. 5:**
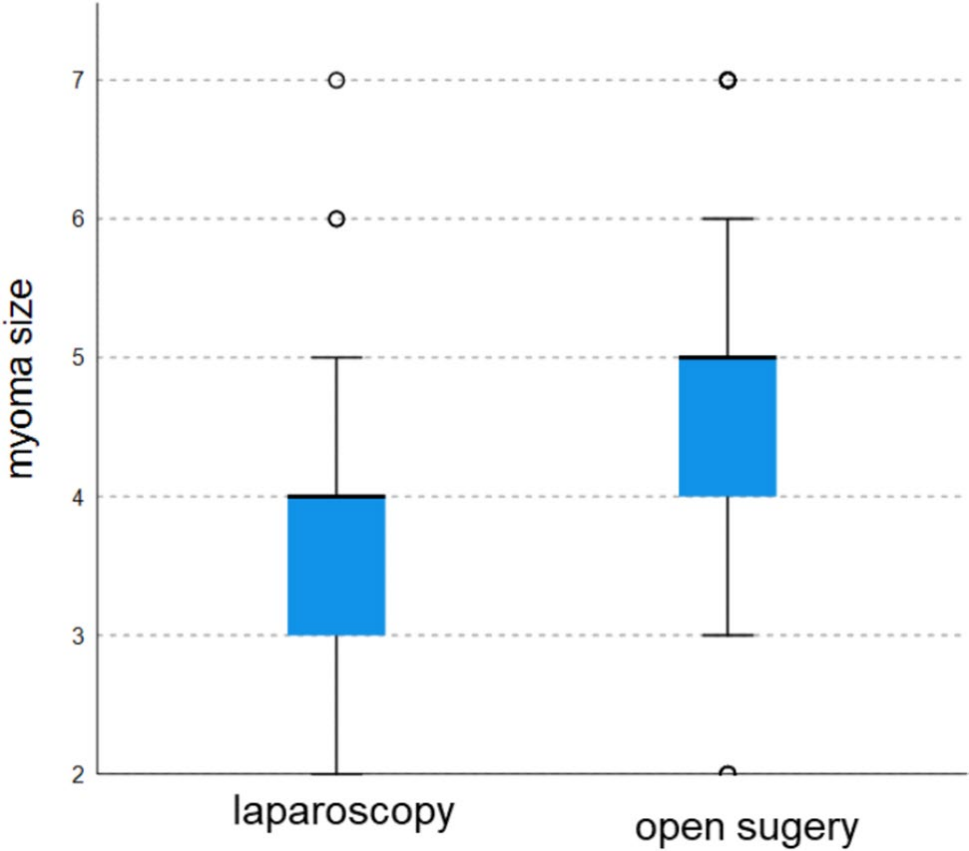
Distribution of myoma size laparoscopic versus open surgery

53% of patients had only a single myoma (56.4% in laparoscopy, 47.8% in laparotomy) (Fig. 2). In the laparotomy group, there were more women with over two fibroids compared to the laparoscopic group (Fig. 3). Regarding myoma size, the majority were between 5–6 cm (34.5%) (Fig. 4). Myomas in the laparotomy group were larger (15.8% in laparoscopy vs. 6.0% in laparotomy for 1–2 cm; 10.9% in laparoscopy vs. 26.9% in laparotomy for 7–8 cm) (Fig. 5). Most myomas were intramural, with no significant differences between the groups (Table 1).

**Table 1:** Myoma characteristics

	Minimal invasive		Open		Total		Sig.
	%	*n*	%	*n*	%	*n*	
Number of fibroids							
1	56.4	57	47.8	32	53.0	89	*p* = 0.03
2	23.8	24	10.4	7	18.5	31	
3	7.9	8	7.5	5	7.7	13	
4	1.0	1	6.0	4	3.0	5	
5	5.0	5	11.9	8	7.7	13	
6	3.0	3	3.0	2	3.0	5	
7	1.0	1	7.5	5	3.6	6	
8	1.0	1	0	0	0.6	1	
9	0	0	0	0	0	0	
10	1	0	4.5	3	1.8	3	
n/a	1.0	1	1.5	1	1.2	2	
Fibroid size							
1–2 cm	15.8	16	6.0	4	11.9	20	*p* = 0.000
3–4 cm	27.7	28	14.9	10	22.6	38	
5–6 cm	38.6	39	28.4	19	34.5	58	
7–8 cm	10.9	11	26.9	18	17.3	29	
9–10 cm	2.0	2	7.5	5	4.2	7	
> 10 cm	1.0	1	16.4	11	7.1	12	
n/a	4.0	4	0	0	2.4	4	
FIGO							
0	1.1	1	0	0	0.6	1	*p* = 0.76
1–2	7.4	7	8.1	5	7.1	12	
3	3.2	3	8.1	5	4.8	8	
4	28.4	27	40.3	25	31.0	52	
5–6	35.8	34	37.1	23	33.9	57	
7	17.9	17	3.2	2	11.3	19	
8	6.3	6	3.2	2	4.8	8	
n/a	1.1	1	0	0	6.5	11	
Recurrence							
No	89.8	88	76.2	48	81.5	136	*p* = 0.02
Yes	10.2	10	23.8	15	14.9	25	
n/a					4.2	7	

### Postoperative outcomes

After laparoscopy, there was a lower risk of recurrence compared to laparotomy (10.2% vs. 23.8%, *p* = 0.02). No significant difference was observed in recurrence risk based on myoma size and number (*p* = 0.128 and *p* = 0.779). A higher rate of cesarean section recommendations postoperatively was noted in the laparotomy group (55.4% overall, 36.6% in laparoscopy, 86.6% in laparotomy, *p* = 0.000). The laparoscopy group had a slightly shorter period of postoperative impairment in daily life without statistical significance (*p* = 0.286). No significant differences were observed in postoperative complications. In the laparoscopy group, 82.2% of patients reported no complications (pain, hematoma, others), compared to 86.6% in the laparotomy group (*p* = 0.623).

Overall, 26.7% of patients reported no improvement in symptoms, 10.7% reported milder pain, 26.7% reported milder bleeding, and 35.7% reported improvement in both. A significant difference was observed, particularly in bleeding symptoms. In the laparoscopy group, 54.5% reported improvement in bleeding, while in the laparotomy group, 74.6% reported improvement (*p* = 0.002).

Overall, patients were satisfied with the intervention without significant difference between both groups (*n* = 91 laparoscopic approach; *n* = 62 open approach (*p* = 0.587). The scar size was significantly smaller in the laparoscopy group (*p* = 0.000) without impact on patient`s satisfaction (*p* = 0.805). Patients in the laparoscopy group stayed significantly shorter (*p* = 0.000) (Table 2).

**Table 2:** Postoperative outcomes

	Minimal invasive		Open		Total		Sig.
	%	*n*	%	*n*	%	*n*	
Impairment in everyday life							
No	15.0	15	13.8	9	14.5	24	*p* = 0.286
Yes, 1–2 weeks	46.7	52	38.5	25	46.7	77	
Yes, 3–4 weeks	20.0	20	29.2	19	23.6	39	
Yes, > 5 weeks	13.0	30	18.5	12	15.2	25	
Complications							
No	82.2	83	86.6	58	83.9	141	*p* = 0.623
Yes, pain	5.0	5	1.5	1	3.6	6	
Yes, hematoma	2.0	2	3.0	2	2.4	4	
Yes, other	10.9	11	9.0	6	17.0	10	
Complaints							
No improvement	34.7	35	14.9	10	26.8	45	*p* = 0.002
Improvement pain	45.5	46	46.3	31	45.8	77	
Improvement bleeding	54.5	55	74.6	50	62.5	105	
Satisfaction							
No	9.9	10	7.5	5	8.9	15	*p* = 0.587
Yes	90.1	91	92.5	62	91.1	153	
Scar size							
To 5 cm	97.0	98	16.4	11	65.7	109	*p* = 0.000
5–10 cm	1.0	1	61.2	41	25.3	42	
> 5 cm	1.0	1	20.9	14	9.0	15	
Hospital stay							
1–2 days	66.3	67	17.9	12	47.0	79	*p* = 0.000
3–4 days	28.7	29	56.7	38	39.9	67	
> 5 days	5.0	5	25.4	17	13.1	22	

Post surgery 75.4% (*n* = 126) did not conceive, 6% (*n* = 10) experienced at least one miscarriages, and 18.6% (*n* = 31) experienced alive birth. There was no difference in the mode of delivery. There was no significant difference in the pregnancy rate regarding the surgical approach (*p* = 0.227) (Table 3).

**Table 3:** Pregnancy outcomes

	Minimal invasive		Open		Total		Sig.
	%	*n*	%	*n*	%	*n*	
Pregnancy outcome							
No pregnancy	76.0	76	76.6	50	75.4	126	*p* = 0.802
Abortion	5.0	5	7.5	5	6.0	10	
Life birth	19.0	19	17.9	12	18.6	31	
Duration until pregnancy							
< 6 month	15.8	3	16.7	2	16.1	5	*p* = 0.227
6–12 month	10.5	2	33.3	4	19.4	6	
> 1 year	36.8	7	8.3	1	25.8	8	
> 1 year and recurrend abortion	36.8	7	41.7	5	38.7	12	

## Discussion

Due to the availability of various treatment possibilities for uterine myoma comprising myoma embolization, HIFU, hormonal treatments and surgical excision and a lack of comparing data regarding the treatment safety in case of postoperative pregnancies and recurrence rate, the decision making process for the optimal myoma therapy is challenging. The present study aimed to compare open and laparoscopic myomectomy with a focus on impacting quality of life through patient-reported outcomes and pregnancy rates.

Notably, half of the study cohort had no intention or desire to conceive demonstrating the patients' request for a uterus-preserving surgical procedure regardless of their fertility plans. The surgical method had no influence on the pregnancy rate, which was equal in the two study cohorts. The choice between open and laparoscopic surgery involves multiple factors, including patient characteristics (size, number of myomas, previous surgeries, BMI) and surgeon-related factors (experience, morcellation capability) [22–26]. Considering limited large randomized studies with very heterogeneous patient collectives comparing these methods underscore the importance of evaluating clinical data. In the present study cohort, myoma size and presence of multiple myomas were associated with a higher likelihood of undergoing laparotomy.

One complicating aspect in the context of uterus-preserving surgery is the differential diagnosis of a uterine sarcoma. Despite considerable attempts, it is not yet possible to reliably differentiate a benign myoma from a sarcoma preoperatively based on clinical and imaging parameters. However, the accidental diagnosis of uterine sarcomas in the context uterus-preserving surgery is rare. The frequency is reported differently in the literature and ranges between 1/204 and 1/7400 (0.49–0.014%). A summarised analysis of the frequency of accidentally operated uterine sarcomas in 10 international studies with *n* = 8753 operations showed a frequency of 0.24. A meta-analysis of *n* = 10,120 patients from 9 studies showed a comparable frequency of accidentally operated uterine sarcomas of 0.29% [27, 28]. Several retrospective analyses have shown that in the case of a postoperative diagnosis of uterine sarcoma, intra-abdominal morcellation means a worse prognosis for the affected patient [29, 30]. In this context, however, there are also data that show no negative influence, at least with regard to overall survival [31]. The extent to which the negative prognostic factor can be relativized by morcellement in a bag has not yet been investigated [32]. Of note, there was no diagnosis of accidental uterine sarcoma in the study population.

In terms of postoperative outcomes, laparoscopy was associated with a significantly lower recurrence risk compared to laparotomy. This finding might be attributable to the more challenging initial surgical situation which favoured an open surgical approach. These data are also very interesting, as it is often postulated that open surgery may potentially offer better palpation and complete resection of fibroids [7]. However, lower recurrence rates following laparoscopic myomectomy have been also reported by Tsiampa et al. [33]. The laparotomy group had a higher rate of cesarean section recommendations versus the laparoscopy group, likely attributable to larger and more numerous myomas in this group which aligns with existing literature [24, 34]. However, this further points out to the fact that open surgery did not necessarily provide better protection against cavity opening or enable better identification and removal of myomas.

In the literature, an overall advantage is evident for the laparoscopic approach, particularly concerning postoperative morbidity [25, 34, 35]. In the present study, however, postoperative morbidity (postoperative impairment in daily life, postoperative satisfaction, scar size) were numerically slightly worse in the laparotomy cohort but did not show significant differences compared to the laparoscopic approach. Regarding pregnancy outcomes, a surprisingly high proportion (75.4%) of patients did not conceive postoperatively, with most proportion conceiving after 1 year. This aspect should be incorporated into the preoperative counselling. There was no statistical difference between the groups. Existing literature suggests a potentially higher pregnancy rate after laparoscopy of 28 up to 70% [19, 35].

A critical aspect of the analysis is the inherent differences between the groups in preoperative criteria (myoma size and number) influencing the choice of surgical approach. Myoma size and number can also be strong factors affecting operative outcomes independent of the surgical approach. This potential imbalance may have led to a deterioration in outcomes for the open surgery group. Regarding pregnancy outcomes, it is important to consider the small patient sample, and the 2023-operated patients may have had a short follow-up period, potentially affecting the data negatively.

The limitations of the study include the absence of randomized allocation to the individual groups, differences in myoma number and size between the groups, and the relatively short follow-up period for some of the patients especially concerning pregnancy outcomes.

In summary, both surgical procedures demonstrated high patient satisfaction, symptom improvement, and similar pregnancy outcomes. However, a preference for laparoscopy was observed concerning the recommendations for cesarean section and the risk of recurrence. Additionally, there was a tendency towards slightly greater operative satisfaction and fewer complications with laparoscopic procedures.
